# Linoleic acid metabolite leads to steroid resistant asthma features partially through NF-κB

**DOI:** 10.1038/s41598-017-09869-9

**Published:** 2017-08-29

**Authors:** Lipsa Panda, Atish Gheware, Rakhshinda Rehman, Manish K. Yadav, B. S. Jayaraj, SubbaRao V. Madhunapantula, Padukudru Anand Mahesh, Balaram Ghosh, Anurag Agrawal, Ulaganathan Mabalirajan

**Affiliations:** 1 Molecular pathobiology of respiratory diseases, Council of Scientific & Industrial Research (CSIR)-Institute of Genomics and Integrative Biology, Mall Road, Delhi, 110007 India; 2 Department of Biochemistry, JSS Medical College, Jagadguru Sri Shivarathreeshwara University, Mysuru, 570015 Karnataka India; 3 Department of Pulmonary Medicine, JSS Medical College, Jagadguru Sri Shivarathreeshwara University, Mysuru, 570015 Karnataka India

## Abstract

Studies have highlighted the role of nutritional and metabolic modulators in asthma pathobiology. Steroid resistance is an important clinical problem in asthma but lacks good experimental models. Linoleic acid, a polyunsaturated fatty acid, has been linked to asthma and glucocorticoid sensitivity. Its 12/15–lipoxygenase metabolite, 13-S-hydroxyoctadecadienoic acid (HODE) induces mitochondrial dysfunction, with severe airway obstruction and neutrophilic airway inflammation. Here we show that HODE administration leads to steroid unresponsiveness in an otherwise steroid responsive model of allergic airway inflammation (AAI). HODE treatment to allergic mice further increased airway hyperresponsiveness and goblet metaplasia. Treatment with dexamethasone was associated with increased neutrophilic inflammation in HODE treated allergic mice; unlike control allergic mice that showed resolution of inflammation. HODE induced loss of steroid sensitivity was associated with increased p-NFkB in mice and reduced GR-α transcript levels in cultured human bronchial epithelia. In summary, HODE modifies typical AAI to recapitulate many of the phenotypic features seen in severe steroid unresponsive asthma. We speculate that since HODE is a natural metabolite, it may be relevant to the increased asthma severity and steroid insensitivity in patients who are obese or consume high fat diets. Further characterization of HODE induced steroid insensitivity may clarify the mechanisms.

## Introduction

Linoleic acid, a dietary polyunsaturated fatty acid (PUFA), and its lipid metabolites are known to mediate several inflammatory pathways in asthma. Dietary intake of ω-6 and ω-3 fatty acids determines the lipid composition of the cell and its membrane, which in turn affects the cell health. It has been well established that while ω-6 fatty acid and its metabolites are pro-inflammatory, ω-3 fatty acids are majorly anti-inflammatory^1^. Previous studies have shown a positive association between dietary components rich in ω-6 fatty acid such as margarine and vegetables oils (soy, safflower, sunflower and corn), and asthma prevalence^2–8^.

Asthma, which was initially thought to be a Th2 dominant disease, is now considered a heterogeneous syndrome with respect to clinical phenotypes and treatment responses^9, 10^. Among which, obese-asthma (lacks Th2 biomarker) and neutrophil dominant asthma phenotype represent a significant proportion in asthma and respond poorly to corticosteroid treatment^11, 12^. Although, steroid resistance is seen in only 5–10% of the asthmatic population, it consumes significant health resources and contributes to substantial mortality and morbidity^13^. Despite being a major hindrance to the treatment, very little is understood of steroid resistance phenotype and its molecular regulators. The lacunae in the understanding of the mechanism could be partially attributed to the lack of good experimental model. Currently available steroid resistant mouse models are induced by using various external triggers or insults such as OVA and House dust mite^14–17^. However, there are very limited studies with endogenous factors, present upstream of the phenotype observed in steroid resistance. In this context, studying the role of endogenous factors (e.g, lipid metabolites) present in asthmatics may provide better insights into the molecular mechanisms underlying steroid resistance in humans and would be more clinically relevant.

Linoleic acid (ω-6 fatty acid), is known to increase the levels of cytokines which leads to neutrophilia^18^. These are also known to negatively modulate the binding of synthetic glucocorticoids with glucocorticoid-receptor^19, 20^. Its 12/15–lipoxygenase metabolite, 13-S-hydroxyoctadecadienoic acid (hereafter written as HODE), is not only increased in asthmatic lungs but also induces mitochondrial dysfunction, severe airway obstruction with neutrophilic inflammation in naïve mice^21^. Role of dietary lipids and its metabolites is not known in steroid resistance. Hence, in this study, we explored the involvement of HODE in the development of steroid resistance like features of asthma.

Here, we demonstrate for the first time, the importance of a metabolic intermediate, HODE, in the development of steroid resistance. We further provide evidence to support the role of NF-κB and GR-α in the HODE induced steroid insensitivity.

## Results

### Airway inflammation induced by 13-S-HODE, a dietary lipid metabolite, is resistant to steroid treatment

**Figure 1:**
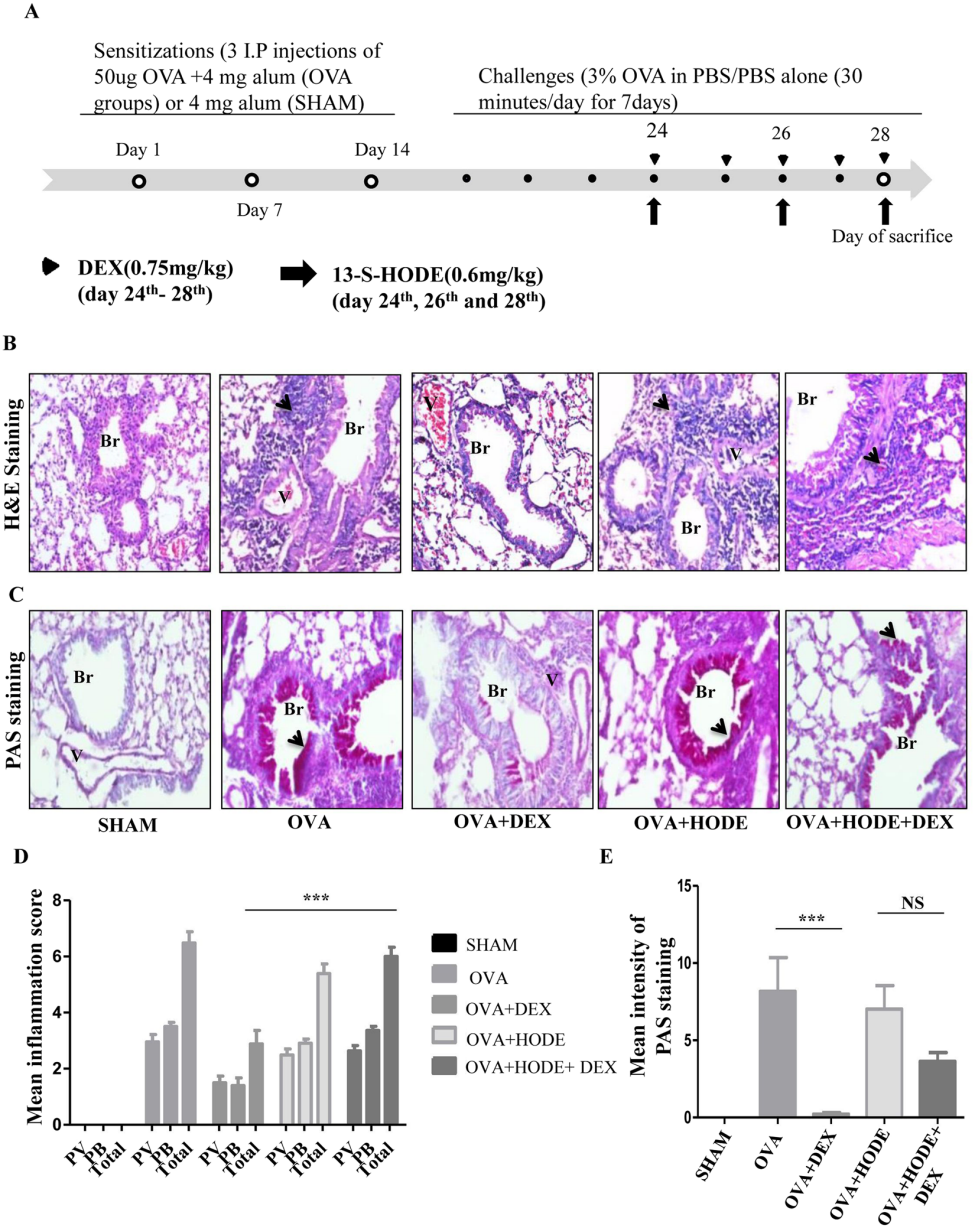
Dexamethasone fails to attenuate airway inflammation and goblet cell metaplasia induced by HODE in allergic mice. (**A**) Schematic representation of experimental design/protocol as described in methods. (**B** and **C**) Representative photomicrographs (20 X magnifications) of bronchovascular regions of different groups of mice stained with haematoxylin and eosin (H & E) and periodic acid–Schiff (PAS). Arrows indicate the infiltrated inflammatory cells in (**B**) and goblet cell metaplasia in (**C**). Mean inflammation score (**D**) and mean intensity of mucin content (**E**) estimated from the images of H and E and PAS stained lung sections. Data represents mean ± SE; n = 4–6 each group; ***p < 0.001, NS, non-significant (OVA versus OVA + HODE + DEX), Br: bronchi, V: vessel.

To investigate the effect of HODE on steroid resistance, HODE (0.6 mg/kg or 2.02 mM) was administered to OVA induced allergic mice intranasally (Fig. 1A). As compared to SHAM, OVA induced mice showed increased infiltration of inflammatory cells and goblet cell metaplasia (GCM), which were alleviated with DEX (dexamethasone, a steroid) treatment. However, DEX was unable to reduce inflammatory cell infiltration and GCM in HODE administered allergic mice (OVA + HODE + DEX) (Fig. 1B–E).

**Figure 2:**
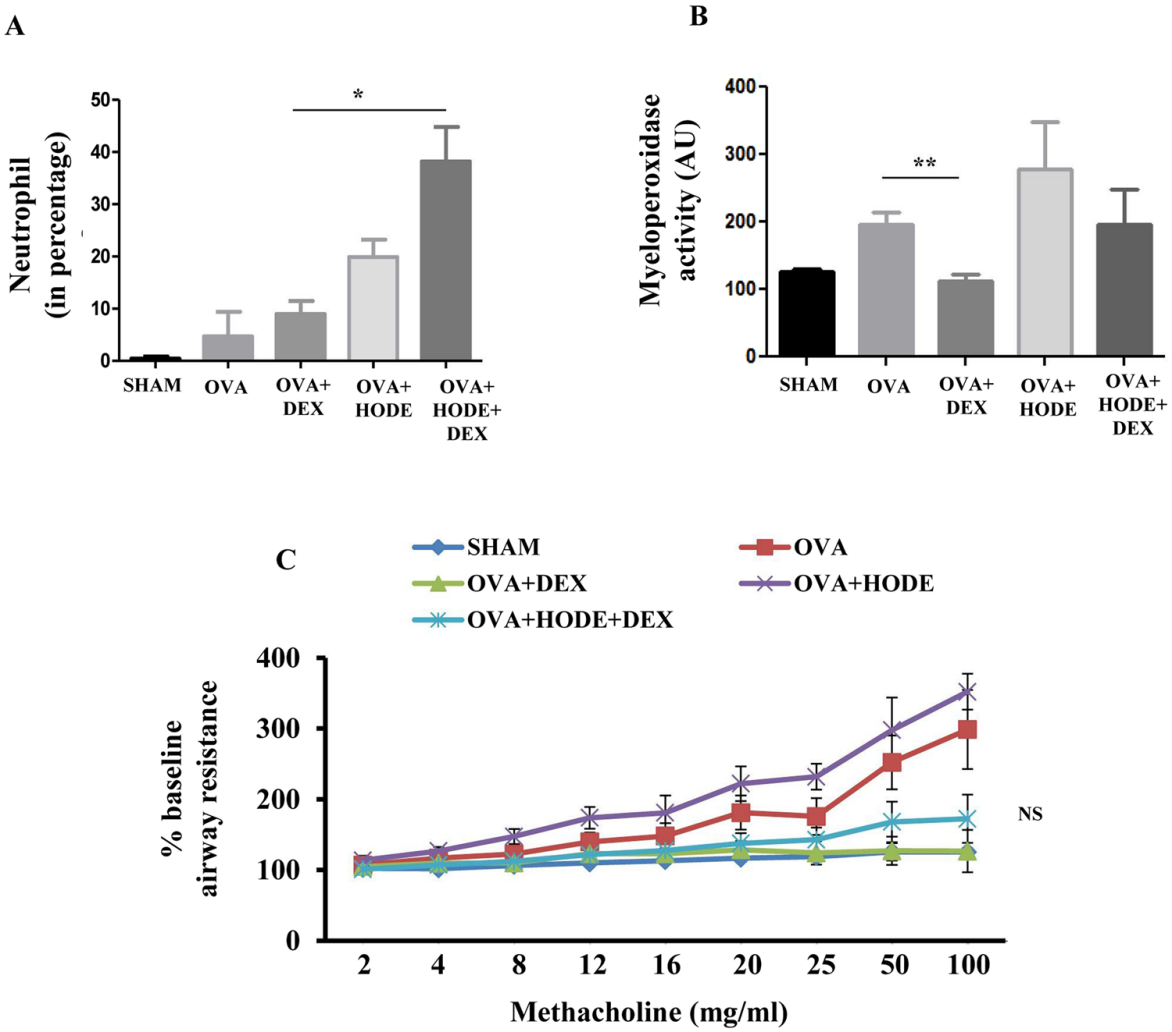
Dexamethasone fails to alleviate HODE induced neutrophilic inflammation and AHR in allergic mice. (**A** and **B**) Neutrophil percentage and myeloperoxidase activity in BAL fluid. (**C**) The percentage baseline airway resistance in response to increasing concentrations of methacholine in HODE administered allergic mice. Data represents mean ± SE; n = 4–6 mice each group; *p < 0.05, **p < 0.01, NS, non-significant (OVA versus OVA + HODE + DEX).

In bronchoalveolar lavage (BAL) fluid, HODE-treated allergic airway inflammation (AAI) mice showed significant increase in eosinophils, lymphocytes and neutrophils. Treatment with DEX was able to reduce the percentage of eosinophils, but not neutrophils (Fig. 2A). In fact, it increased the number of neutrophils in HODE-treated AAI mice. Myeloperoxidase assay suggested that the active neutrophils were significantly reduced by DEX treatment in OVA mice, but not in HODE-treated OVA mice (Fig. 2B). Further, DEX could not reduce airway hyper-responsiveness (AHR) in response to methacholine in OVA mice administered with HODE (OVA + HODE + DEX) when compared to OVA alone mice (OVA + DEX) (Fig. 2C).

### HODE administration reduced GR-α and its activity in human bronchial epithelial cells

**Figure 3:**
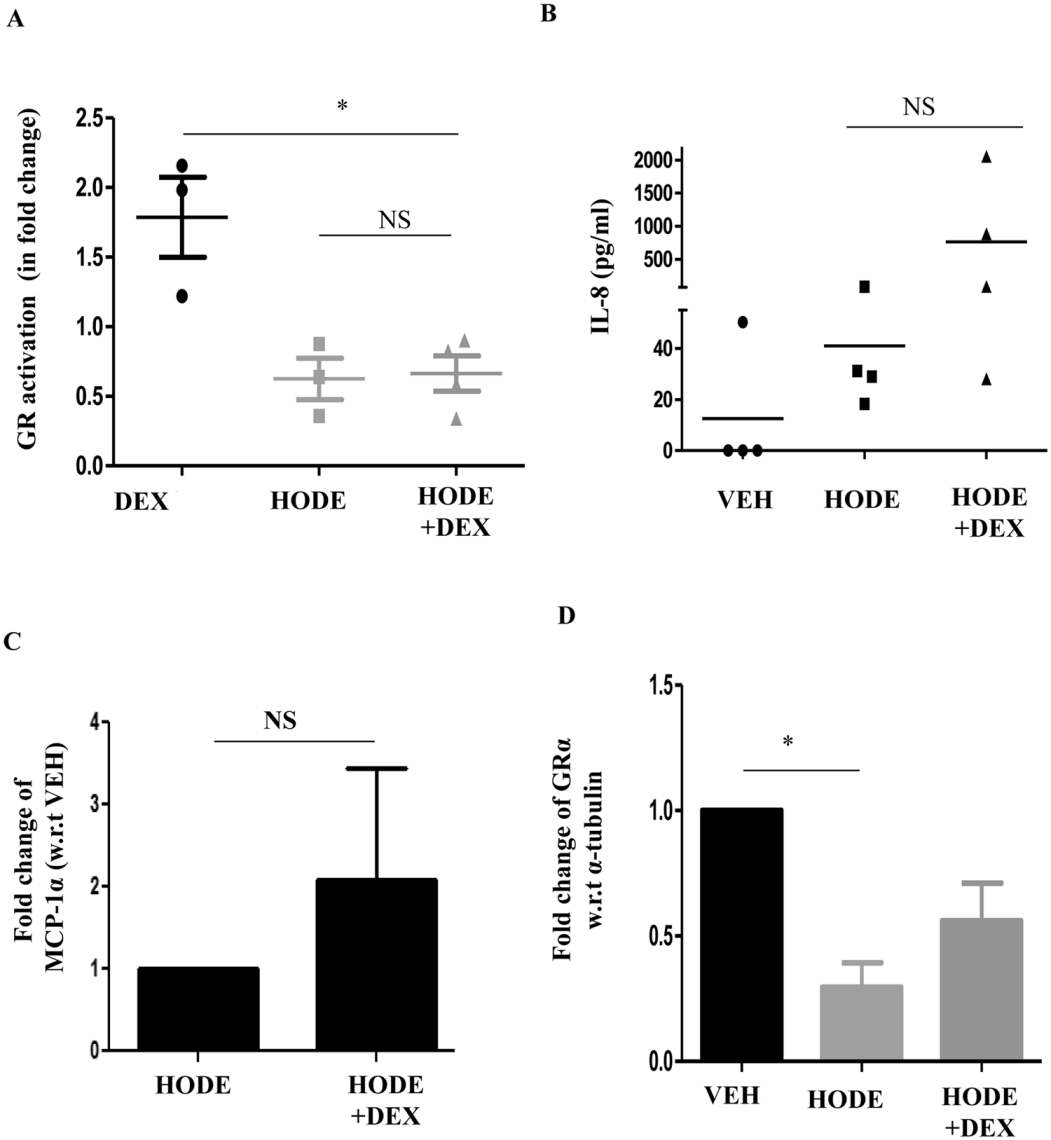
HODE reduces GR-α and its activation in BEAS-2B cells. (**A**) Glucocorticoid receptor (GR) activation was estimated in nuclear extract of HODE induced Beas2B cells (details in methods). (**B** and **C**) Levels of IL-8 and MCP1-α in the supernatants of cultured human bronchial epithelia, induced with HODE and DEX. (**D**) Transcript levels of GR-α normalized to α-tubulin. Data represents mean ± SE; n = 3–5; *p < 0.05, NS: non-significant.

To determine whether HODE-mediated steroid resistance was through direct effects on the glucocorticoid response, we studied the effect of HODE on glucocorticoid receptor. Glucocorticoid response is mediated by glucocorticoid receptor (GR) that binds to glucocorticoid response element (GRE) and modulates the expression of the downstream genes^22^. To examine whether HODE affects GR activation, binding of GR to synthetic GRE oligonucleotides was estimated in nuclear extracts of dexamethasone pretreated bronchial epithelia (BEAS-2B), which were induced with HODE. HODE reduced the GR activation when compared to dexamethasone alone, and this reduction was not restored with addition of dexamethasone (Fig. 3A). Downstream effects of dexamethasone, such as suppression of IL-8 and MCP-1α, were also abolished by HODE (Fig. 3B,C). To determine whether HODE-mediated reduction in GR activation was due to decrease in the GR-α receptor expression, we measured the transcript levels of GR-α in BEAS-2B cells. HODE treatment led to a significant decrease in the levels of GR-α expression (Fig. 3D).

### HODE induced inflammation upregulated p-NFκB in allergic mice

**Figure 4:**
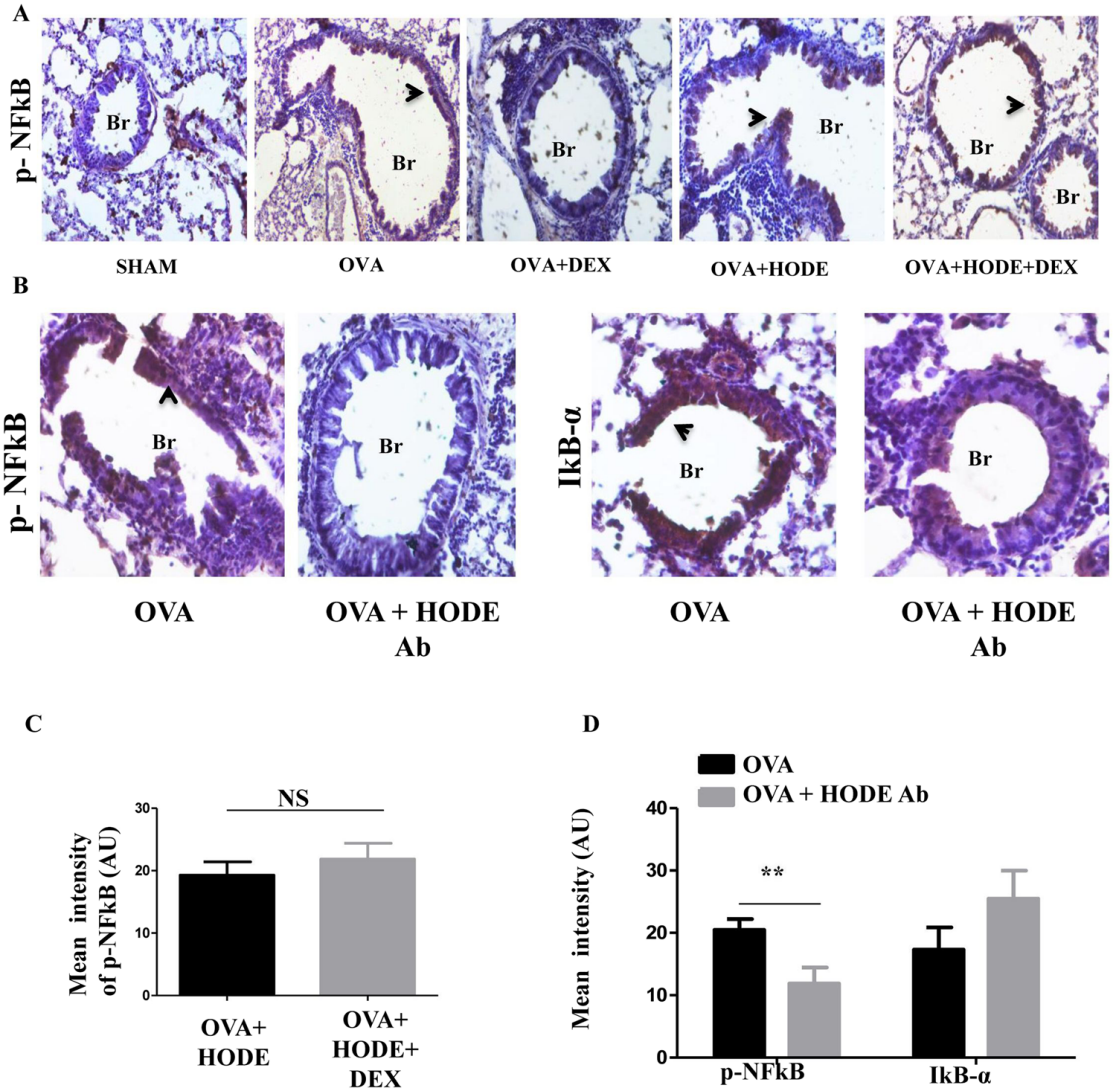
HODE administration increases p-NFκB in AAI mice. (**A** and **C**) Representative IHC images (20X magnifications) and quantification of the expression of p-NFκB in HODE induced steroid resistance model. (**B** and **D**) Representative IHC images (20X magnifications) and quantification of p-NFκB and IκB-α in the lung sections of HODE neutralized allergic mice. Data represents mean ± SE; n = 3–6; **p < 0.01, NS: non-significant. Br: Bronchi. Arrows indicate the positive expression

To determine whether HODE-mediated steroid resistance is via the transient receptor potential cation channel subfamily V member 1 (TRPV1), which may mediate HODE-induced asthma like features^21^, we knocked down TRPV1 in our steroid resistant model. However, siRNA mediated knock down of TRPV1 did not resolve AHR, AAI or MPO activity in this model (Supplementary Fig. 1). Since NF-κB activation is previously reported in steroid resistant asthma^23^, we next performed immunohistochemical measurement of p-NFκB p65 (Ser 536). We found that OVA challenge led to a DEX-sensitive p-NFκB increase in airway epithelium. HODE treatment was associated with lack of p-NFκB decline post DEX treatment. HODE neutralization was associated with a significant reduction in p-NFκB, along with a trend towards restoration of IκBα levels (Fig. 4A–D).

### Inhibition of NF-κB alleviated HODE induced steroid resistant inflammation in allergic mice

**Figure 5:**
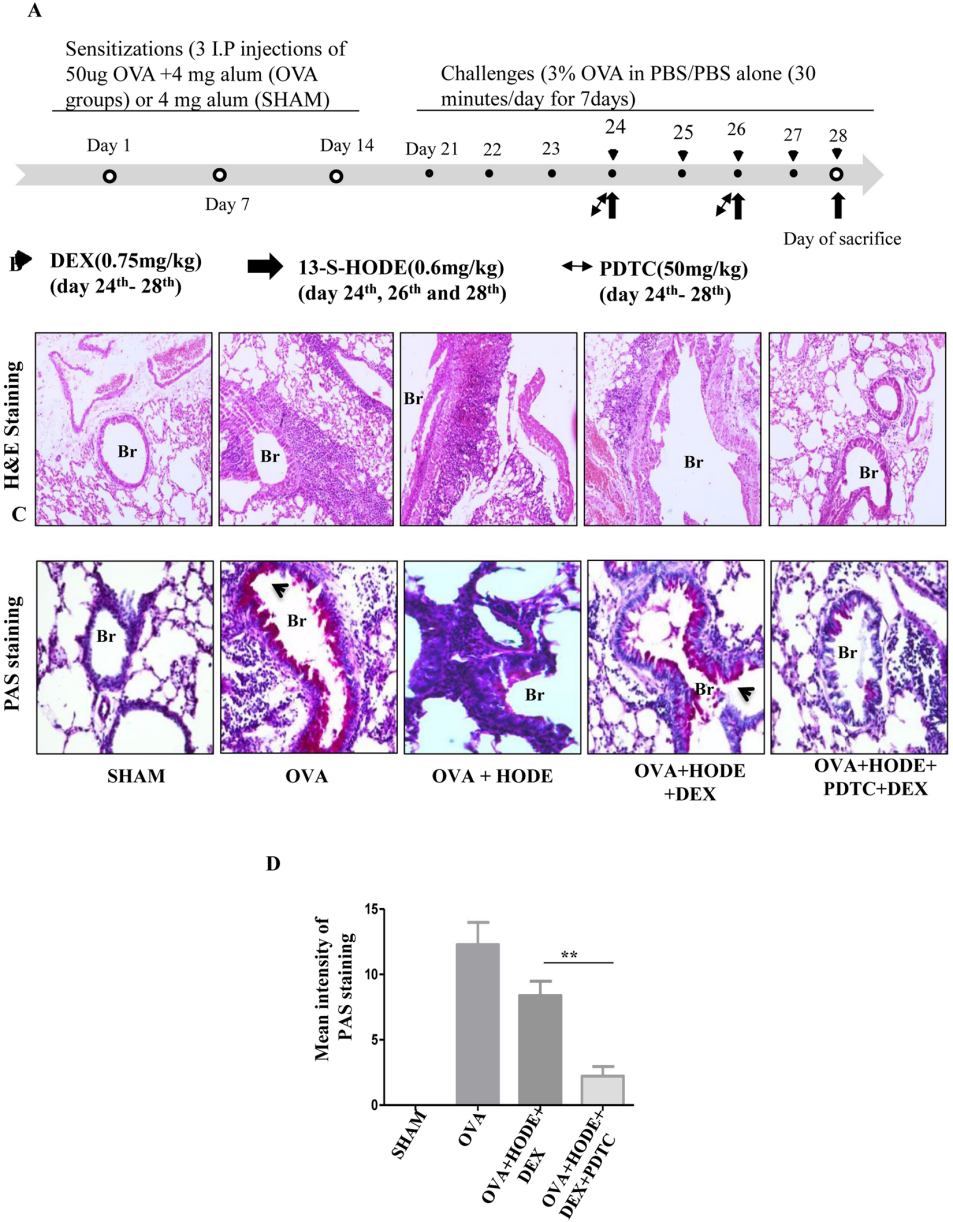
NFκB inhibitor treatment increases steroid sensitivity, reduces airway inflammation and goblet cell metaplasia in HODE induced steroid resistant mice. (**A**) Schematic representation of experimental design. PDTC (50 mg/kg) was administered intraperitoneally on day 24^th^ and day 26^th^, 2 hrs and 4 hrs after the administration to HODE and DEX respectively (details explained in material and methods). (**B** and **C**) The representative photomicrographs of H and E (20x magnifications) and PAS, respectively. (**D**) Mean intensity of PAS calculated by Image J software. Data represents mean ± SE; n = 3–6; **p < 0.01, NS: non-significant. Br: bronchi, V: vessel, Arrows indicate the goblet cell metaplasia.

**Figure 6:**
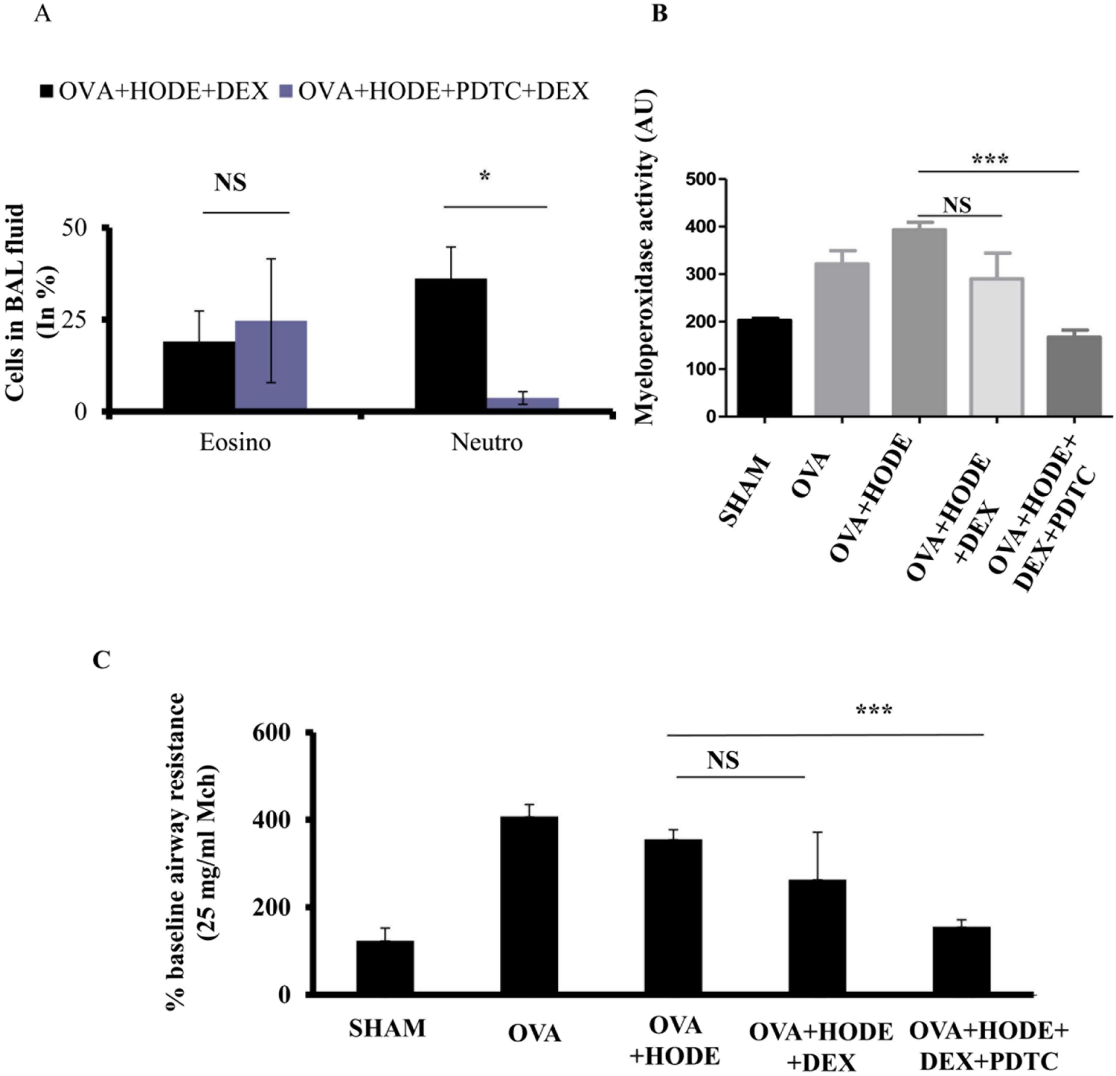
NFκB inhibitor treatment alleviates neutrophilic inflammation and airway hyperresponsiveness in HODE induced steroid resistant mice. (**A**) The counts of neutrophils and eosinophils in BAL fluid of steroid resistant mice administered with PDTC. (**B**) The myeloperoxidase activity in mouse BAL fluid supernatants. (**C**) The percentage baseline airway resistance in response to 25 mg/ml methacholine dose. Data represents mean ± SE; n = 3–6 each group; *p < 0.05,***p < 0.001, NS: non-significant.

To verify the role of NF-κB in HODE mediated steroid resistance, we tested whether the steroid resistance was reversible by pyrrolidinedithiocarbamate (PDTC, 50 mg/kg), a potent NF-κB inhibitor (Fig. 5A). PDTC increased the sensitivity to DEX in HODE-treated steroid resistant mice. Infiltration of inflammatory cells as well as GCM were reduced in lung sections of PDTC treated OVA + HODE + DEX mice compared to OVA + HODE + DEX mice (Fig. 5B–D). Cells in BAL fluid indicated that PDTC specifically reduced neutrophilic airway inflammation. We also found a significant reduction in myeloperoxidase activity in PDTC treated OVA + HODE + DEX mice, when compared to HODE-treated OVA mice (Fig. 6A,B). PDTC administered mice also showed reduced AHR in response to 25 mg/ml methacholine than HODE-treated OVA mice (Fig. 6C).

## Discussion

While there are numerous reports on the effect of dietary lipids and its metabolites on the pathogenesis and increased incidence of asthma^2–8^, there are no studies indicating its role in steroid resistant asthma. We, for the first time, show that 13-S-HODE, the 12/15 LOX metabolite of linoleic acid, leads to steroid resistance. This was shown in mice with AAI and in cultured human bronchial epithelial cells. OVA induced AAI mice, which are typically sensitive to steroids, showed steroid resistant airway hyperresponsiveness and goblet cell metaplasia upon HODE induction. In human bronchial epithelial cells, HODE led to decreased GR-α transcript. GR-α mediates the effects of glucocorticoids by further binding to positive or negative glucocorticoid response elements that mediate activation or repression of downstream genes, respectively^22^. Decrease in the transcript levels of GR-α, is expected to lead to reduced activity of steroids and hence, steroid insensitivity. GR-β, the decoy receptor of GR-α, has also been studied in steroid resistant asthma patients. These studies suggest an increase in the expression of GR-β in the neutrophils^24–27^. However, in our study, we did not find any significant changes in the expression of GRβ in HODE induced bronchial epithelial cells (data not shown). As for the murine model, the presence of GRβ and its role in steroid resistance remains equivocal. It has been demonstrated using *in-vitro* cellular systems that unsaturated fatty acids like linoleic acid negatively modulate the binding of triamcinolone acetonide or dexamethasone, a synthetic glucocorticoid, with glucocorticoid-receptor^19, 20^. With respect to the current study, it would be interesting to investigate the direct or indirect mechanism with which HODE could modulate GR-α.

There are numerous metabolites derived from linoleic acid through its downstream fatty acids like gamma linolenic acid, and arachidonic acid and certain enzymes like Δ6-desaturase, elongase, Δ5-desaturase, lipoxygenases, cyclooxygenase and cytochrome P450^28^. Many of the linoleic acid metabolites like leukotoxin, isoleukotoxin, leukotrienes are pro-inflammatory mediators^29^. Though 5-lipoxygenase/leukotrienes pathway are known to be crucial in causing bronchospasm, the role of 15-lipoxygenase and its downstream metabolites were not well explored. In this context, we have shown the involvement of 15-LOX and its metabolites in causing mitochondrial dysfunction in asthma pathogenesis^30, 31^. Though there are numerous metabolites of linoleic acid,we focused on 13-S-HODE as we found highly increased levels of this in asthmatic patients in our earlier study^21^. As we have demonstrated the involvement of IL-4/IL-13/15-LOX pathway on mitochondrial dysfunction^21, 31^, we were interested to see the effects of 13-S-HODE, a downstream metabolite of this pathway on airway function and further steroid resistance. It would be interesting to study the effects of linoleic acid rich diet on the airway function and steroid resistance. These supplementation studies have to demonstrate the levels of linoleic acid and its metabolites in the airway before dissecting the role of linoleic acid diet supplementation on steroid resistance as asthma predominantly affect the airways in general. In this context, it has been shown that linoleic acid supplementation indeed worsens the cystic fibrotic conditions with increased levels of pro-inflammatory mediators like IL-8^32^. So it would be interesting to see the effects of high fat especially linoleic acid diet rich diet on steroid resistance. In any event high fat fed mice had shown the steroid resistant features (Singh VP *et al*., unpublished data from our lab).

Our group has administered intranasal 13-S-HODE to demonstrate its relation with severe asthma. We indeed calculated the intranasal dosage from the 13-S-HODE levels present in the BAL fluids of human asthmatics in reference to IL-4 and IL-13 levels in BAL fluids of human and mice asthmatic conditions as 13-S-HODE is the product IL-4 and IL-13 signaling. It has been shown in a number of studies that though the BAL fluid concentrations of IL-4 and IL-13 do not differ at basal conditions in asthmatic conditions, it reaches up to 200–400 pg/ml after allergen challenge^33–35^. To mimic IL-4 or IL-13 mediated human relevant asthmatic condition in mice, 3–5 µg of recombinant IL-4 or recombinant IL-13 per day has been widely used^36, 37^. So we have used 15 µg of 13-HODE to approximately 25 gram mouse (0.6 mg/kg) as we found approximately 1200 pg/ml of 13-S-HODE in the BAL fluids of human asthmatics^21^.We did not check the levels of HODE in blood of mice that were administered intranasal HODE and so we are not sure whether intranasal HODE reached bloodstream or not. But there is a good possibility that it might spill over to bloodstream as we administered HODE in the OVA induced inflamed lungs. In addition, we found increased levels of endogenous HODE in sera of human asthmatics^21^.

**Figure 7:**
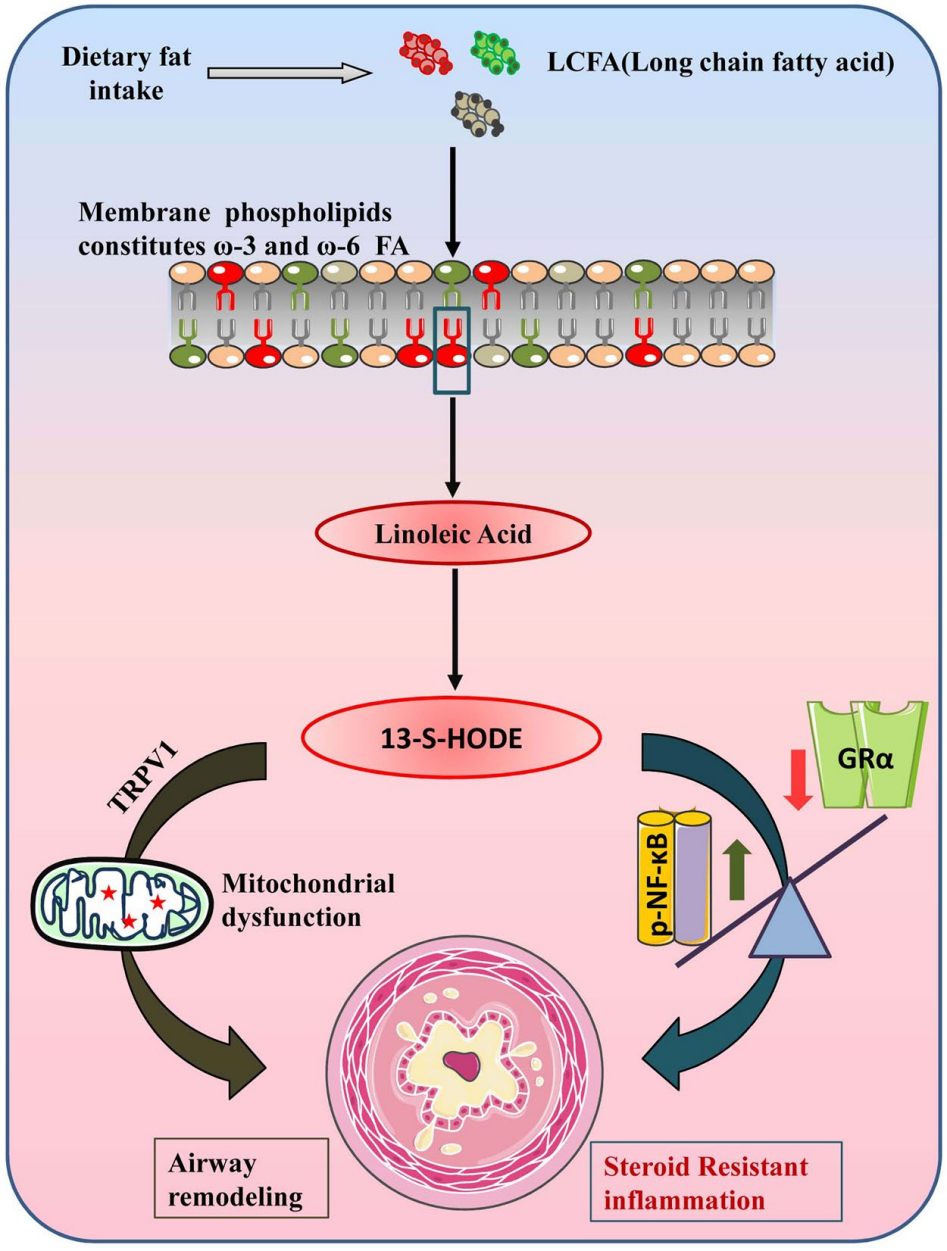
13-S-HODE mediates steroid resistance via NF-κB and GR-α. Dietary lipids, absorbed through intestine are converted to long chain fatty acids which serve as a precursor for the formation of phospholipids. While, ω-6 fatty acids (red color phospholipid) are oxidized into pro-inflammatory lipid mediators, ω-3 is (green color phospholipid) oxidized to anti-inflammatory. 13-S-HODE is a lipid metabolite of linoleic acid (ω-6 fatty acid), oxidized by 15-lipoxygenase. 13-S-HODE, causes airway remodeling and goblet cell metaplasia by mitochondrial dysfunction via TRPV1 channels. In another independent pathway, it increases p-NFκB and reduces GR-α leading to steroid resistant asthma.

Our previous report demonstrates that naïve mice treated with HODE had increased neutrophilia and high Th17 cytokines, which are often seen in patients with steroid resistant asthma^12, 13^. Though the chemotactic activity of 13-hydroxy-linoleic acid on the isolated exogenous neutrophils is known^38^, we have shown the *in vivo* demonstration of HODE induced airway neutrophilia. In the present study, HODE induced steroid resistance in mice with AAI was not associated with any change in IL-17A, IL-21 or IL-22 (data not shown), suggesting that there may be different mechanisms of action of HODE in uninflamed and inflamed lungs. This is supported by our observation that TRPV1 inhibition, which attenuates the effects of HODE in naive mice^21^, had no effect in the steroid resistant AAI model. Also, it would be interesting to investigate the effects of DEX in HODE administered naïve mice as this could reveal the possible effects of DEX on non-allergic steroid resistant inflammatory conditions with increased levels of 13-S-HODE. However, in human bronchial epithelial cells, DEX treatment in presence of HODE was unable to reduce the steroid responsive cytokines such as IL-8 and MCP-1α. While IL-8 and MCP-1α are steroid regulated cytokines, these cytokines are also known to be regulated by NF-κB. And interestingly, GR-α inhibits NF-kB mediated pro-inflammatory cytokines by physically interacting with p65 subunit (Rel-A), creating a competition for the binding of coactivators and preventing the phosphorylation of RNA polymerase II^39^. In this scenario, the reduced GR-α in HODE induced BEAS-2B implies the loss of inhibition of GR-α on NF-κB, thereby increasing the expression of cytokines driven by NF-κB. There are substantial reports suggesting the involvement of NF-κB in multiple inflammatory pathways of asthma, some of which also converge with steroid mediated pathways. Similar to these, we also observed that the loss of steroid sensitivity in mice was associated with increased p-NFκB. NF-κB is known to regulate the expression of cytokines such as KC (mouse homologue of IL-8) and G-CSF which helps in neutrophil chemotaxis and survival. We show that pyrrolidinedithiocarbamate (PDTC) administration specifically inhibited the neutrophilic inflammation in HODE induced steroid resistant mice and increased the sensitivity towards steroids, therefore, resolving the steroid resistant features. Although, we clearly observed that HODE induced steroid resistance was due to activated NF-kB, we did not find any significant change in the transcript levels of RelA/p65 in HODE induced BEAS-2B cells (data not shown). Thus, the mechanisms underlying HODE mediated NF-kB activation are yet to be investigated.In any event, the interplay of reduced GR-α and increased p-NFκB thus appears to be critical in the development of HODE-induced steroid resistance (Fig. 7).

In the present study, we have focused only on the NF-kB pathway. However, the involvement of other pathways relevant to steroid resistance like ERK1/2, JNK, and p38 mitogen-activated protein kinase signaling pathways^40^ in this steroid resistance model needs to be explored. So, the existence of NF-κB independent mechanisms to regulate the expression of GR has to be investigated in details. However, we envisage the involvement of nuclear receptors regulated by lipid metabolites. Also, it would be interesting to compare the levels of lung HODE among steroid resistant and steroid sensitive patients, although the levels of HODE are known to be increased in sera of asthmatic patients. We speculate that such studies will lead to a greater mechanistic understanding of steroid resistance in asthma and clarify the role of dietary lipids with respect to steroid sensitivity. There is an increase in the dietary ω-6/ω-3 fatty acid ratio due to the westernization of food consumption patterns^41^. This gradual change in the fatty acid composition of the diet, including an increase in the level of ω-6 fatty acids and ω-6/ω-3 fatty acid ratio with time is associated with increased risk and prevalence of obesity^41^, which emerges as a risk factor for asthma development^42–45^. Obese-asthma phenotype requires greater deal of attention and studies as it is typically refractive to steroid treatment^43, 45^ and this is where we speculate our study can bridge the gap. Moreover, it would be interesting to check the levels of HODE in obese-asthmatic patients and correlate it with the steroid responsiveness to further strengthen the hypothesis. Our study would help in understanding the pathogenesis of steroid resistance, overarching all the phenotypes including obese-asthmatics.

## Methods

### Mice grouping

The male BALB/c mice (6–8 weeks) were procured from Central Drug Research Institute, Lucknow, India and maintained in Institute of Genomics and Integrative Biology (IGIB), Delhi, India. The Institutional Animal Ethical Committee at IGIB approved all mice experiments and all methods were performed in accordance with the relevant guidelines and regulations of Committee for the Purpose of Control and Supervision of Experiments on Animals (CPCSEA). Two different allergic models were utilized; first being, OVA model with five groups: SHAM (mice that were PBS sensitized, PBS challenged and treated with vehicle, 50% ethanol), OVA (mice that were OVA, grade V chicken egg ovalbumin, sensitized, OVA challenged and treated with vehicle), OVA + DEX [allergic mice treated with dexamethasone (0.75 mg/kg) orally], OVA + HODE [allergic mice administered with intranasal HODE (0.6 mg/kg or 2.02 mM) and treated with vehicle] and OVA + HODE + DEX [allergic mice administered with intranasal HODE (0.6 mg/kg or 2.02 mM) and treated with dexamethasone (0.75 mg/kg) orally]. The second model had following groups: SHAM, OVA, OVA + DEX, OVA + HODE + DEX, and OVA + HODE + DEX + PDTC [OVA + HODE + DEX mice administered with PDTC dissolved in DMSO (50 mg/kg) intra-peritoneally].

### OVA-immunization and challenge

Mice were sensitized with three intraperitoneal injections of 50 µg OVA adsorbed in alum for three weeks and challenged with 3% OVA in PBS for 7 days as described earlier^21, 46, 47^.

### Administration of 13-S-HODE, Dexamethasone, and PDTC

13-S-HODE (Cayman, Michigan,USA) or VEH (50% ethanol) was instilled to the nasal openings of each isoflurane anesthetized mouse. Based on our previous publication^21^ we have selected the dose of 0.6 mg/kg or 2.02 mM for each mouse. 13-S-HODE was administered intranasally on days 24, 26 and 28 as shown in Fig. 1A. Dexamethasone (Sigma-Aldrich, MO, USA), dissolved in 50% ethanol, was given orally (0.75 mg/kg) to mice from day 24 to 28 as shown in Fig. 1A. PDTC (Sigma-Aldrich, USA) was dissolved in DNAase and RNAase free H_2_O, and was administered intraperitoneally into mouse (50 mg/kg) on days 24, 26 and 28, 2 hrs after the administration of HODE (Figs 1A and 5A).

### Airway hyperresponsiveness measurement and bronchoalveolar lavage (BAL)

Airway hyper-responsiveness was estimated with invasive flexiVent (SCIREQ, Montreal, Canada) as previously described^21, 46, 47^. BAL was performed and differential cell counts were made as described earlier^21, 46, 47^.

### Lung histopathology

Formalin-fixed lung sections were stained with Haematoxylin & Eosin (H & E), Periodic acid-Schiff and morphometric analysis was performed using publicly available Image J software^21, 46, 47^.

### *In vitro* experiments

Human bronchial epithelial cells (Beas-2B) were obtained (ATCC, Manassas, VA, USA), maintained in HAM’s F12 (Sigma-Aldrich, MO, USA) with 10% fetal bovine serum (FBS). The cells were pretreated with dexamethasone (10^−6^ M, Sigma, MO, USA) for 3 hrs before stimulating with vehicle (ethanol) or 13-S-HODE (35 μM, Cayman, Ann Arbor, Michigan, USA) for 16 hrs. These cells were harvested for further experiments and supernatants were stored for cytokine assays.

### ELISAs

IL-8, MCP1-α (E-biosciences, CA, USA), myeloperoxidase assay (Cayman chemicals, Michigan, USA) were performed according to manufacturer’s instructions from culture supernatants (IL-8, MCP1-α) and BAL Fluid respectively.

For measuring GR activity (Active Motif, CA, USA) BEAS-2B cells obtained after HODE and DEX treatment (as described above), were processed for nuclear extract. Elisa was performed using manufacturer’s protocol with 20 μg of nuclear extract. The O.D measured is plotted in arbitrary units (AU) in fold change, calculated by O.D test/O.D veh^48^.

### Immunohistochemistry

Immunohistochemical analysis for p-NFkB and IkB-α (Santa Cruz Biotechnology, Texas, USA) was performed with respective secondary antibodies (Sigma, St. Louis, MO, USA).

### Real Time PCR

Cells harvested were lysed in RNA lysis solution and RNA was isolated (Qiagen, Germany). cDNA was isolated from RNA using the manufacture’s protocol (ABI, CA, USA). Further real time was performed using kappa FAST Syber green (Roche cycler) using the following primers. Human GR-α, FP: 5′ACGGTCTGAAGAGCCAAGAG3′and RP: 5′CAGCTAACATCTCGGGGAAT3′; Human β-actin, FP: 5′CCAACCGCGAGAAGATGA3′, RP: 5′ CCAGAGGCGTACAGGGATAG3′.

### Statistical analysis

All data represents mean ± SEM; n = 3–6 each group; *p < 0.05, **p < 0.01, ***p < 0.001. A p-value more than 0.05 is considered non-significant (NS). Statistical significance of the differences between paired groups was determined with a two-tailed Student’s *t* test. One-way analysis of variance was used to compare multiple groups by using PRISM software and was evaluated further with a nonparametric Mann-Whitney rank-sum test or Krusker-wallis test wherever appropriate.

## Electronic supplementary material

Supplementary info
