# Supplementary material for: Linoleic acid metabolite leads to steroid resistant asthma features partially through NF-κB
Source: Sci Rep. 2017 Aug 29;7:9565. doi: 10.1038/s41598-017-09869-9 (PMC5575291; doi:10.1038/s41598-017-09869-9)
Supplement: Supplementary file 1 — Supplementary info [file 41598_2017_9869_MOESM1_ESM.pdf]

Linoleic acid metabolite leads to steroid resistant asthma features partially through NF-κB

Lipsa Panda,[a] Atish Gheware,[a] Rakhshinda Rehman,[a] Manish K. Yadav,[b] BS Jayaraj,[c] Madhunapantula V. SubbaRao,[b] Padukudru Anand Mahesh,[c] Balaram Ghosh,[a] Anurag Agrawal, ,[a] Ulaganathan Mabalirajan[a].

[a]Molecular pathobiology of respiratory diseases, Council of Scientific & Industrial Research (CSIR)-Institute of Genomics and Integrative Biology, Mall Road, Delhi-110007, India.

Departments of Biochemistry[b] and Pulmonary Medicine[c], JSS Medical College, Jagadguru Sri Shivarathreeshwara University, Mysuru-570015, Karnataka, India.

Correspondence: Ulaganathan Mabalirajan, Molecular pathobiology of respiratory diseases, CSIR-Institute of Genomics and Integrative Biology, Mall Road, Delhi-110007, India. Phone: 91-11-27662580. Fax: 91-11-27667471, 91-11-27416489. E-mail ID: mabsome@yahoo.co.in

This work was supported by the projects BSC 0116, MLP 5502 (CSIR), GAP 0118 (Department of Biotechnology) and GAP0084 (Department of Science & Technology) at Institute of Genomics & Integrative Biology, Council of Scientific and Industrial Research, Govt. of India.

Disclosure of potential conflict of interest: The authors have declared that they have no conflict of interest.

Fig. S1

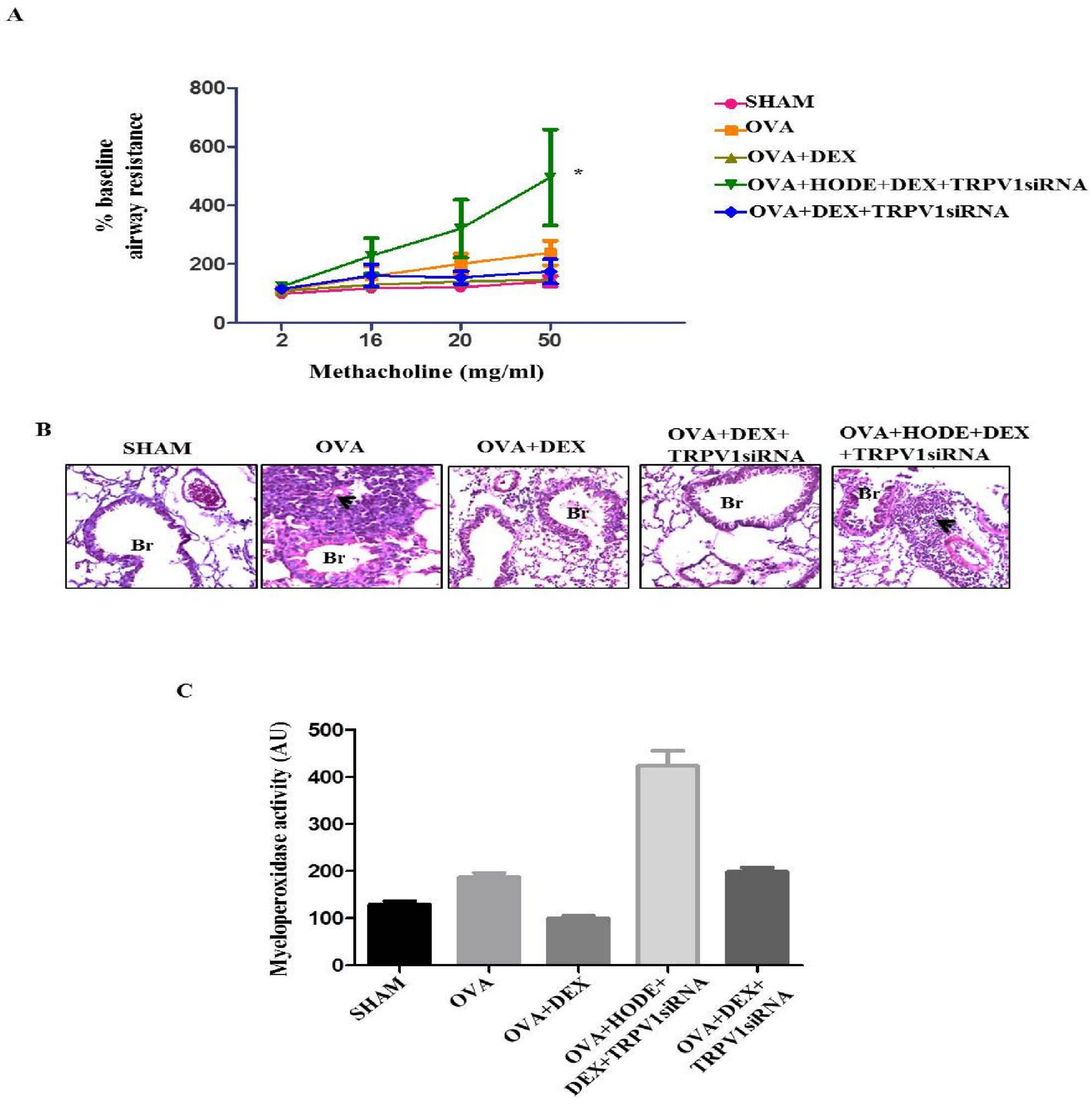

Supplementary Fig 1. HODE mediated steroid resistance is not mediated by TRPV1

A) The percentage baseline airway resistance in response to increasing concentrations of methacholine, **B)** Representative photomicrographs (20 X magnifications) of bronchovascular regions of different groups of mice stained with haematoxylin and eosin (H & E) , **C)**

Myeloperoxidase activity in BAL Fluid of TRPV1 knockdown steroid resistant mice.Data represents mean ± SE; n= 3-6 each group; *p <0.05 (OVA+HODE+TRPV1siRNA+DEX versus OVA+DEX+TRPV1siRNA), Br: bronchi.
